# Optimal techno-economic assessment of isolated microgrid integrated with fast charging stations using radial basis deep learning

**DOI:** 10.1038/s41598-024-70063-9

**Published:** 2024-09-04

**Authors:** Abdelmonem Draz, Ahmed M. Othman, Attia A. El-Fergany

**Affiliations:** Electrical Power and Machines Department, Zagazig University, Zagazig, 44519 Egypt

**Keywords:** Fast charging stations, Electric vehicles, Renewable energy sources, Energy storage systems, Microgrids, Energy management strategies, Electrical and electronic engineering, Energy grids and networks

## Abstract

The global transportation electrification commerce sector is now booming. Stakeholders are paying an increased attention to the integration of electric vehicles and electric buses into  the transportation networks. As a result, there is an urgent need to invest in public charging infrastructure, particularly for fast charging facilities. Consequently, and to complete the portfolio of the green environment, these fast-charging stations (FCSs) are designed using 100% of renewable energy sources (RESs). Thus, this paper proposes an optimization model for the techno-economic assessment of FCSs comprising photovoltaic and wind turbines with various energy storage devices (ESDs). In this regard, the FCS performance is evaluated using flywheels and super capacitors due to their high-power density and charging/discharging cycles and rates. Then, optimal sizing of these distributed generators is attained considering diverse technical and economical key performance indicators. Afterwards, the problem gets more sophisticated by investigating the effect of RES’s uncertainties on the selection criterion of the FCS’s components, design and capacity. Eventually, as an effort dedicated to an online energy management approach, a deep learning methodology based on radial basis network (RBN) is implemented, validated, and carried out. In stark contrast to conventional optimization approaches, RBN demonstrates its superiority by obtaining the optimum solutions in a relatively short amount of time.

## Introduction

### Motivation

**Figure 1:**
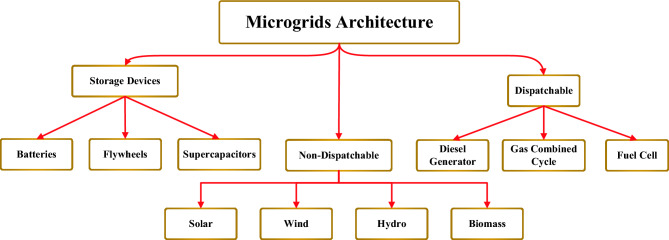
Architecture of Microgrids.

**Figure 2:**
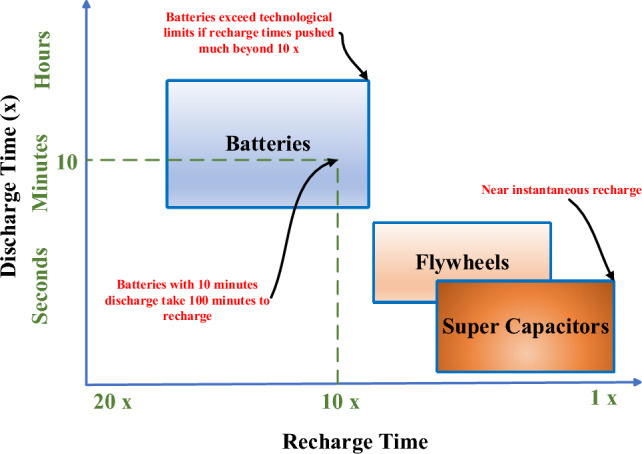
Charging /discharging characteristics of ESDs.

Negative environmental impacts of fossil fuel sources besides their high energy costs are considered as the main motivators for developing sustainable energy^1,2^. In order to minimize carbon emissions and operating costs, micro grids (MGs) are equipped with energy management systems which perform economic dispatch and unit commitment processes^3,4^. MGs utilize the concept of decentralized generation in which the load demand is met by various types of renewable energy sources (RESs) and energy storage devices (ESDs)^5–7^. Distributed generators (DGs) as revealed in Fig. 1 can be classified as dispatchable sources when the generation is controlled to meet the demand or non-dispatchable when the generation is uncontrolled. Non-dispatchable DGs are weather-dependent sources that are intermittent in nature which in turn brings out the need for installing ESDs such as batteries or super capacitors (SCs)^8,9^. It is worth mentioning that the selection of non-dispatchable sources relies on meteorological data such as temperature, solar radiation, and wind speed^10,11^. It is worth noting that flywheels and SCs are characterized by fast discharging rates as declared in Fig. 2 that make them the favorable options in fast charging stations (FCSs) due to their high-power density. Moreover, the usage of batteries will not be applicable if the recharge time exceeds a certain limit as revealed in Fig. 2.

Due to the emissions produced by conventional gasoline vehicles, they are replaced by electric vehicles (EVs) as an environmentally friendly solution^12,13^. However, the deployment of EVs fleet across roadways attracts the attention of utility operators for the implementation of public charging infrastructures^14^. FCSs represent the widespread solution in highways for customer’s satisfaction^15,16^ although they bring technical and economic issues^17^. Power quality, voltage stability, and overloading problems are samples of the technical challenges facing the utility’s planners^18^. This is due to the fact that FCSs are rapacious burdens on the electric network because of demanding huge power in very short time duration^19,20^. FCSs may be deemed as hybrid renewable microgrid (HRMG) comprising various forms of ESDs operating either in an on-grid mode or off-grid mode to complete the portfolio of sustainable environment^21^.

### Literature survey

A novel framework is introduced in^22^ for the optimal energy management in MGs in which the spatial temporal of energy exchange between EVs is considered. Moreover, with the aid of V2G technology, the charging price and dispatch are optimized using the chance constrained optimizer along with the deep Q-learning network. Utilizing the principles of electricity time of use in addition to real time pricing in^23^ are beneficial in the demand side management of MG with different types of RESs. A combination of batteries and SCs is exploited in^24^ to regulate the voltage in DC MG regardless of the intermittent nature of RESs or load variations. Excess electricity problem or the unused surplus power in hybrid renewable off-grid networks is investigated in^25^ using various approaches which aid in the development of this MG configuration. It is worth noting that technical, economic, environmental, and social constraints are incorporated in the optimization framework addressed in^26^ for energy dispatch in HRMG supplying residential and telecommunication loads.

The investigated methodology in^27^ deals with stand-alone HRMG network comprising thermal energy storage systems (ESSs). The obtained results manifest the superiority of implementing recover exhaust heat system over the baseline scenario without any thermal energy storage. In addition, a generalized model based on the demand response program is employed in^28^ for minimizing the MG operating cost and CO2 emissions. Afterwards, employing data driven programming with multilayer perceptron in restoring the non-linearity feature in energy conversion components is explored. The proposed methodology in^29^ with the aid of load forecasting techniques enhances the sustainability of the HRMG system by determining the accurate capacities of DGs. Fuzzy-based forecasting followed by multi-criteria decision approach is utilized in ranking the optimal solutions considering diverse performance indicators.

Multi-objective optimization algorithms are interrogated in^30^ for optimal allocation and sizing of DGs with battery storage system (BSS) to reinforce the voltage stability and lessen the yearly expenses. In^31^, the formulation of configuration optimization model is proposed to reduce the investment cost using multiple forms of ESDs. In^32^, various optimizers are discussed for optimal designing of HRMG considering technical, environmental, and economical objectives. In this context, the net present cost $\left(NPC\right)$, loss of power supply probability (LPSP), and greenhouse gas $\left(GHG\right)$ emissions are deemed as the main aspects in this multi-objective optimization framework. Smart energy management approach in HRMG with BSS is presented in^33^ using the modified frog leaping optimizer for different cases. Furthermore, the performance Chameleon Swarm Optimizer (CSO) is examined in^34^ for optimally design and sizing of stand-alone HRMG minimizing the $NPC$ along with attaining the reliability constraint in terms of $LPSP.$ In this regard, HOMER software is employed in^35^ investigating technical, economical, and social constraints.

Uncertainty in weather conditions have been tackled in^36^ for optimal sizing of grid connected HRMG fulfilling power quality requirements in terms of harmonics mitigation and power factor correction. In this context, particle swarm optimizer (PSO) is utilized in^37^ for voltage enhancement besides power losses alleviation in radial rural electric power grid. In addition, unmet load fraction $\left({UML}_{f}\right)$ constraint is addressed in^38^ using HOMER software achieving the lowest $NPC$ and $GHG$ emissions using various combinations of RESs. Synergy of these forms of RESs is investigated in^39^ for optimal operation strategy of HRMG participating in energy markets: electricity and hydrogen markets. In^40^, diverse control strategies such as load following (LF), cycle charging (CC) are examined to decide the selection between fuel cell (FC) and BSS at each time step to minimize the total $NPC.$

Biological inspired optimizer (BIO) is implemented and compared with various algorithms in^41^ for optimal design of an off-grid wind turbine (WT) comprising hydrogen energy storage (HES) systems. This optimization framework is analyzed with sensitivity analysis based on two objectives namely system cost and load losses. In^42^, a novel energy management strategy with deploying onsite electrolysers and HES systems equipped with photovoltaic (PV) panels is interrogated for supplying FC EVs, while^43^ presents a comprehensive review of the techniques implemented in the proposed dilemma. The optimal design of electric vehicle charging station in^44^ along with techno economic assessment of HRMG in Egypt in^45^ represent gateways in the preparation of this paper. Additionally, components and specifications of FCSs are summarized in^46^ to augment the literature survey of this research.

### Research gap, paper organization, and contribution

Most of the literature deals with the conventional HRMG comprising PV, WTs, and BSS supplying residential, commercial, or industrial loads. In addition, this optimization dilemma is solved using various metaheuristic-based optimizers considering various operational scenarios. In this context, Table 1 announces a brief comparison between various HRMG configurations discussed previously in the literature. It can be highlighted that the MG topology comprises PV, WT, BSS, HES, FC, or diesel generators for typical installed buildings or regions. However, implementation of ESDs such as SCs and flywheels for electrified transportation loads in the energy management dilemma still acquires more attention. Moreover, deep learning-based tools have not been utilized so far in these optimization processes to alleviate the larger computational time of optimization algorithms. Therefore, the contribution of this research can be summarized as follows:

✔ Explore the performance of the HRMG in feeding new pattern of loads represented in EVs and electric buses(EBs).
✔ Investigate the HRMG operation in public transportation networks acting as a FCS.
✔ Incorporate various forms of ESDs such as BSS, SCs, and flywheels to earn the fast-charging feature to the HRMG.
✔ Optimizing the HRMG configuration besides the component’s installed capacity in normal and fast charging operation modes.
✔ Examine the quality of the optimized architecture in terms of different forms of technical and economical key performance indicators (KPIs).
✔ Investigate the effect of uncertainties in renewables resources on the optimized solutions.
✔ Utilizing a novel deep learning radial basis network in determining the operational capacity of the HRMG in online applications.

**Table 1:** Summary of HRMG projects discussed in the literature.

Reference	Year	MG topology	Optimizer	Application	Location	Remarks
^34^	2023	PV/WT/Tidal/BSS/HES	CSO	Rural region in Fuxin	China	Economic and reliability constraints are addressed
^37^	2022	PV/WT/BSS	PSO	Rural grid of Guissia	Cameroon	BSS aids in power factor correction
^38^	2023	PV/WT/CHP/ESS	HOMER	Oakland university	United States	Results demonstrate the economical effectiveness of WT and CHP in off-grid mode
^40^	2022	PV/FC/BSS	HOMER	Students services center building	United States	Interference with MATLAB for control strategy optimization
^41^	2023	WT/HES	BIO	Residential load	China	Various designs of WTs are included
^44^	2022	PV/Diesel/BSS	SSO	Northwest region in Delhi	India	Design of electric vehicle charging station
^45^	2022	PV/WT/BSS/Diesel	HOMER	National research center farm	Egypt	Demand side management participates in peak shaving which in turns the oversizing is avoided
^47^	2021	PV/WT/BSS	GWO	Ras-Shaitan in Sinai	Egypt	Reliability is evaluated using $LPSP$ index
^48^	2024	PV/WT/HES/BSS	HOMER	General	-	Sensitivity analysis of excess electricity
^49^	2024	PV/WT/HES/FC	BIO	Residential building	China	Sensitivity analysis of wind speed and interest rate
^50^	2024	PV/WT/BSS	CPLEX	Chinese Yuan	China	Battery lifetime model is incorporated
^51^	2024	WT/BSS	HSO	Typical power system	China	Various technologies of batteries
^52^	2024	PV/biomass/Diesel/BSS	GPO	New Tiba City	Egypt	Optimal configuration is cropped
^53^	2024	PV/WT/FC/BSS/HES	AVO	Marsa Matrouh	Egypt	V2G technology is considered
^54^	2024	PV/WT/BSS/Diesel	HOMER	Uttara University	Bangladesh	Various aspects have been investigated

*HSO harmony search optimizer, *GPO* gradient pelican optimizer, *AVO* african vultures optimizer.

The organization of current research is summarized as follows; Section “Research Methodology” presents the research methodology for the technical and economic study and analysis, including the configuration, modeling and optimization. Section “Modelling of the HRMG” discusses the system modeling and mathematical representation for the problem statement. Section “Objective Function, Associated Constraints, and KPIs” focuses on the optimization process and formulation, where the fitness function with related constraints and performance indicators are structured. Afterwards, Section “Simulation Results and Discussions” consolidates the numerical analysis with operational results and scenarios. Finally, Section “Conclusions” concludes the work with some highlighting of the simulation results.

## Research methodology

**Figure 3:**
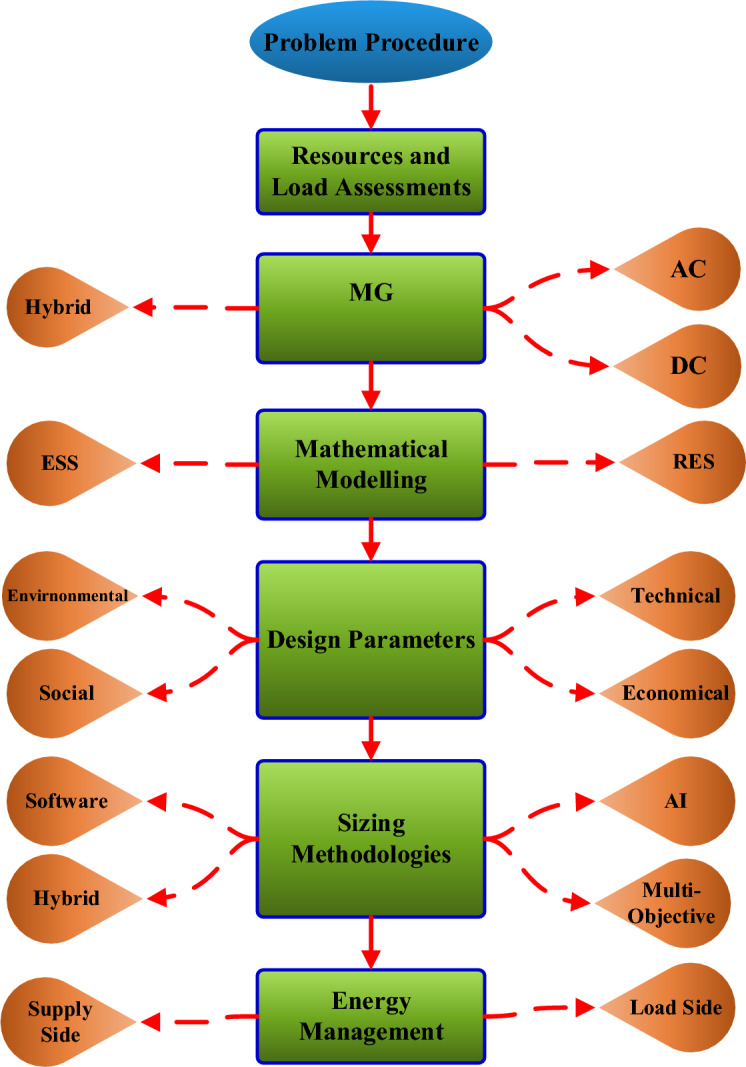
Research Methodology in Steps.

Techno-economic evaluation of HRMGs goes through dedicated steps starting from data collection till results extraction as shown in Fig. 3. These steps can be summarized as follows:

### Resources and load assessments

Assessments of renewable resources availability such as solar irradiation and wind speed are on-site measurements that depend on the project location. For sizing these RESs, load calculations are carried out for diverse categories like AC loads, DC loads, residential, commercial, industrial, and so on.

### MG configuration

Based on the project location, nature, and resources availability, a combination of renewable and non-renewable DGs along with ESDs is implemented for a specific configuration of HRMG. The architecture of HRMG may be classified into three main categories; AC, DC, or hybrid configuration while it may be worked in on-grid or off-grid operating modes.

### Mathematical modelling of HRMG

The power for each unit in the HRMG configuration is mathematically represented and estimated at each simulated time slot. Afterwards, these governing equations are incorporated into the optimization framework based on the selected sizing methodology.

### Design parameters of HRMG

The chosen design parameters are crucial for a more reliable and effective solution. ${UML}_{f}$, $LPSP$, equivalent loss factor, and excess electricity portion $\left(EEP\right)$ are considered as technical constraints. Economical constraints may take various forms such as $NPC$, annualized system cost $\left(ASC\right)$, and cost of energy $\left(CoE\right)$ while GHG represent the widespread environmental factor.

### Sizing methodologies of HRMG

Optimization or artificial intelligence techniques are implemented for optimal sizing and dispatching the generating and storage units. Moreover, multi-objective approach is exploited for this dilemma using pareto or fuzzy decision tools. In addition, commercial software is also employed like HOMER, HYBRIDS, and TRNSYS.

### Energy management in HRMG

Proper energy management is substantial either in load or supply side for reliable and cost-effective operation of HRMG. Load side management comprises different forms like peak shaving, peak shifting, valley filling, and flexible load curve. On the other hand, some rules nominated as dispatch strategies are used to control the operation of generator and ESDs such as LF, CC, generator order, predictive strategy, and combined dispatch.

LF dispatch method operates the generator for load supplying when needed while RESs charge the storage bank. In this context, charging process of ESDs is the least priority in generator’s operation while RESs take over this mission. CC dispatch method enforces the diesel generator to run at its rated capacity regardless of the load value. Therefore, the surplus power is used to charge the storage batteries until they reach the maximum state of charge $\left(SoC\right)$ level. CC dispatch technique is the best candidate whenever the resources of renewables are not adequate.

## Modelling of the HRMG

### Solar PV modelling

**Figure 4:**
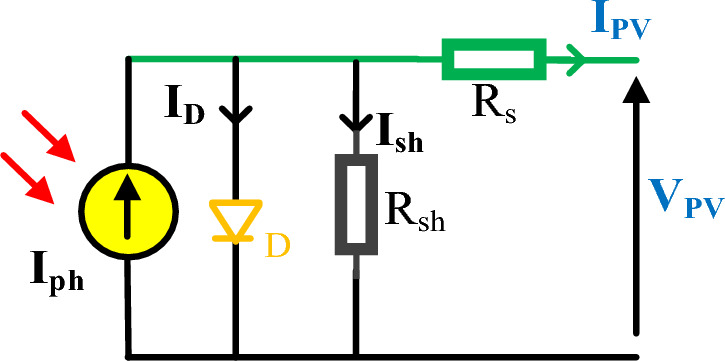
Simplified equivalent circuit of PV model.

**Figure 5:**
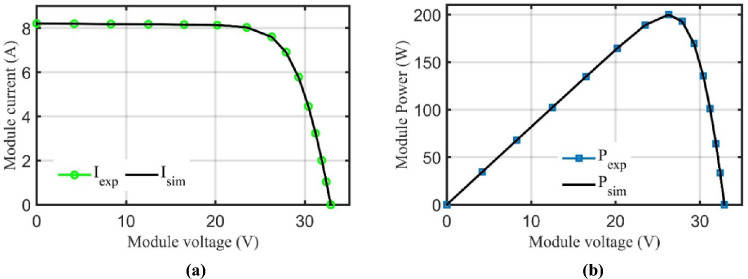
PV cell characteristics. (**a**) Current v.s Voltage and (**b**) Power v.s Voltage.

Generally, the PV is modelled by the equivalent circuit shown in Fig. 4 which consists of the photo-generated current $\left({I}_{ph}\right)$ represented by a current source, diode $\left(D\right)$, series resistance $\left({R}_{s}\right)$, and shunt resistance $\left({R}_{sh}\right)$. In this regard, PV cell performance is evaluated by (current–voltage) and (power-voltage) characteristics as depicted in Fig. 5 with three governing points: open circuit voltage $\left({V}_{oc}\right)$, short circuit current $\left({I}_{sc}\right)$, and maximum power point $\left({V}_{mpp},{I}_{mpp}\right)$. These three points are stamped in the PV datasheet and nameplate which dominate the PV performance under various temperature and solar irradiation. The PV output current ${(I}_{PV})$ may be estimated from (1) by calculating the shunt resistance current ${(I}_{sh})$ and diode current ${(I}_{D})$ from (2) and (3) respectively.

1 $${I}_{PV}={I}_{ph}-{I}_{D}-{I}_{sh}$$

2 $${I}_{sh}=\frac{{V}_{PV}+{I}_{PV}{R}_{s}}{{R}_{sh}}$$

3 $${I}_{D}={I}_{rs}\left({e}^{\frac{{V}_{PV}+{I}_{PV}{R}_{s}}{n{V}_{T}}}-1\right)$$

4 $${V}_{T}=\frac{K{T}_{c}}{q}$$

where ${I}_{rs}$ denotes the diode reverse saturation current, $n$ symbolizes the diode ideality factor, ${V}_{T}$ designates the thermal voltage that is assessed from (4) where $K$ is the Boltzman constant = $1.3806503\times {10}^{-23}$, $q$ is the electron charge = $1.602\times {10}^{-19}C$, and ${T}_{c}$ is the cell temperature.

The clearness index $(CI)$ of the studied zone is estimated from (5) which depends on the portion of horizontal extra-terrestrial solar irradiation ${(G}_{h,av})$ and monthly available solar irradiation $({G}_{av})$. The instantaneous cell temperature $({T}_{c}\left(t\right))$ is computed from (6) where $NOCT$ stands for normal operating cell temperature while $({T}_{amb}\left(t\right), G\left(t\right))$ are the instantaneous ambient temperature and solar irradiation in $\left(W/{m}^{2}\right)$ respectively. In this context, the instantaneous PV output power $({P}_{PV}\left(t\right))$ is calculated from (7) while the total output power ${(P}_{TPV}\left(t\right))$ is calculated from (8) where $({N}_{PV})$ is the number of PV modules.

5 $$CI=\frac{{G}_{av}}{{G}_{h,av}}$$

6 $${T}_{c}\left(t\right)={T}_{amb}\left(t\right)+\left(\frac{NOCT-20}{800}\right)\times G(t)$$

7 $${P}_{PV}\left(t\right)={V}_{PV}\left(t\right)\times {I}_{PV}\left(t\right)={P}_{PV@STC}\left[1+{k}_{p}\left({T}_{c}\left(t\right)-{T}_{@STC}\right)\right].{F}_{PV}.\left(\frac{G(t)}{{G}_{@STC}}\right)$$

8 $${P}_{TPV}\left(t\right)={P}_{PV}\left(t\right)\times {N}_{PV}$$

where ${F}_{PV}$ is the cell derating factor and ${k}_{p}$ is the maximum power temperature coefficient, and ${P}_{PV@STC}$, ${T}_{@STC}$, ${G}_{@STC}$ denote the PV output power, cell temperature, and solar irradiation at Standard test conditions $STC$ (25 ^0^C, and 1000 $W/{m}^{2}$).

### Wind turbine modelling

**Figure 6:**
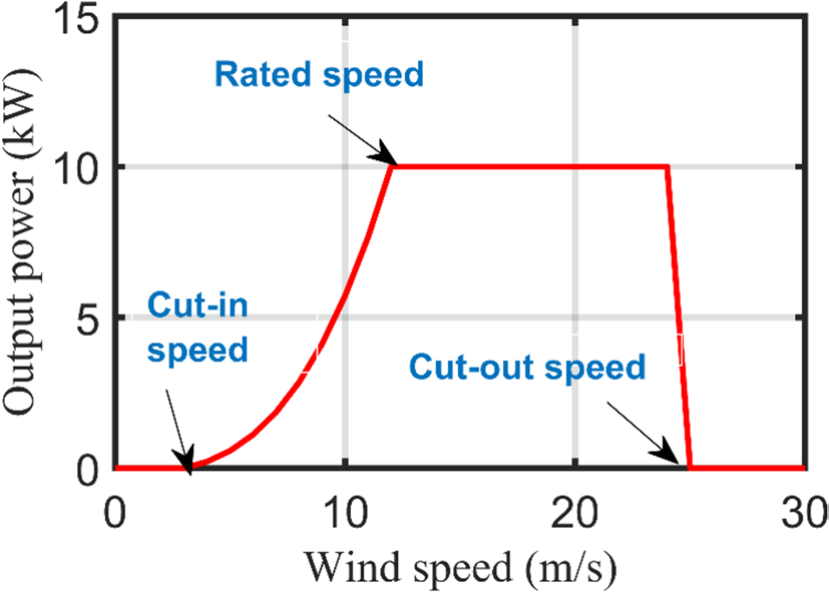
WT power-speed curve.

Each WT has a typical power output curve as depicted in Fig. 6 which describes the relation between the output power and average wind speed. First, the measured wind speed by the anemometer shall be corrected to the hub height location as illustrated in (9).

9 $${V}_{h}={V}_{an}\times {\left(\frac{{h}_{h}}{{h}_{an}}\right)}^{\gamma }$$

where ${V}_{an}$ denotes the measured wind speed at the anemometer height while ${V}_{h}$ is the calculated wind speed at the hub height, ${h}_{an}$ and ${h}_{h}$ are the anemometer and hub height respectively, $\gamma$ is the Hellmann coefficient or the roughness factor that ranges from 0.1 to 0.25 based on the investigated zone.

The generic equation that correlates the instantaneous WT output power $({P}_{WT}\left(t\right))$ with the instantaneous wind speed at hub height $({V}_{h}\left(t\right))$ is given in (10). However, as declared in (11) and Fig. 6, the WT output power can be estimated according to the wind speed for three different operating regions. The output power equals zero when the wind speed is below the cut-in speed ${(v}_{ci})$ or excesses the cut-out speed ${(v}_{co})$. Contrarily, the WT power remains constant at the rated power ${(P}_{r})$ between the rated speed $({v}_{r})$ and ${v}_{co}$ while it varies with the cubicle of wind speed in the region between ${v}_{ci}$ and ${v}_{r}$. Furthermore, the total output power $({P}_{TWT}\left(t\right))$ from ${(N}_{WT})$ units is calculated from (12).

10 $${P}_{WT}\left(t\right)=0.5{\rho }_{a}A{{V}_{h}(t)}^{3}{C}_{p}{\eta }_{o}$$

11 $${P}_{WT}\left(t\right)=\left\{\begin{array}{cc}0& {v}_{(t)}\le {v}_{ci},{v}_{(t)}\ge {v}_{co} \\ {P}_{r}\left(\frac{{v}_{\left(t\right)-}^{3}{v}_{ci}^{3}}{{v}_{r}^{3}-{v}_{ci}^{3}}\right)& {v}_{ci}<{v}_{(t)}<{v}_{r}\\ {P}_{r}& {v}_{r}<{v}_{(t)}<{v}_{co}\end{array}\right\}$$

12 $${P}_{TWT}\left(t\right)={P}_{WT}\left(t\right)\times {N}_{WT}$$

where ${\rho }_{a}$ is the air density $\left(kg/{m}^{3}\right)$, $A$ is the rotor blades swept area, ${C}_{p}$ is the WT power coefficient that varies between 0.3 to 0.5, ${\eta }_{o}$ is the electro-mechanical conversion efficiency, and ${N}_{WT}$ is the number of WT units.

### Batteries modelling

The charging process of the batteries bank is done through the surplus energy which comes from the increment of PV-WT generation at any time t during the simulation process as explained in (13), while in (14), the discharging process of the batteries bank occurs.

13 $${E}_{batt}\left(t\right)={E}_{batt}\left(t-1\right)\left(1-\sigma \right)+\left({P}_{excess}\times {\eta }_{charge}\right)$$

14 $${E}_{batt}\left(t\right)={E}_{batt}\left(t-1\right)\left(1-\sigma \right)-\left(\frac{{P}_{def}}{{\eta }_{discharge}}\right)$$

where ${E}_{batt}\left(t\right), {E}_{batt}\left(t-1\right)$ are the stored energy of battery at time slot t and t-1 respectively, while $\sigma$ denotes the self-discharge rate of the battery. ${P}_{excess}$ is the surplus power generated from RESs over the demand, while ${P}_{def}$ is the deferrable power in which the demand exceeds the generated power from RESs, and ${\eta }_{charge}, and {\eta }_{discharge}$ are charging and discharging efficiencies of the battery, respectively.

Afterwards, the minimum number of storage batteries $({N}_{batt})$ can be evaluated from (15) for more reliable HRMG operation.

15 $${N}_{batt}=\frac{AHC}{{AHC}_{r}}$$

where $AHC$ is the required ampere hour capacity for the reliable operation which evaluated from (16) while ${AHC}_{r}$ is the rated capacity of the selected batteries model.

16 $$AHC=\frac{{E}_{load}\times {n}_{days}}{DoD\times {V}_{B}\times {\eta }_{s}}$$

where ${E}_{load}$ denotes the load daily average energy $\left(kWh\right)$, ${n}_{days}$ denotes the number of days in which the batteries bank is energized, $DoD$ is the maximum depth of discharge, ${V}_{B}$ is the battery voltage, and ${\eta }_{s}$ is the battery-inverter system efficiency. Furthermore, the battery autonomy is computed from (17) which is the ratio between the capacity of the batteries bank and the average daily electric load.

17 $${T}_{batt,aut}=\frac{AHC\times {V}_{B}\times \left(1-{SoC}_{min}\right)}{{E}_{load}\times 1000}$$

The battery lifetime throughput $({E}_{batt,life})$ is the amount of stored energy in $kWh$ that the battery is expected to supply during its life time which can be calculated from (18). Therefore, the storage batteries need to be replaced after a specific number of failure cycles $({N}_{cycles,f})$ as marked in the datasheet (Number of charging and discharging cycles that can be completed before losing performance).

18 $${E}_{batt,life}=\frac{{N}_{cycles,f}\times DoD\times {AHC}_{r}\times {V}_{B}}{1000}$$

### Flywheels modelling

The kinetic energy stored in the rotating mass of the flywheel depends on the angular speed of rotation and moment of inertia as revealed in (19). As described in (20), the stored kinetic energy can be boosted by optimizing the rotor mass and shape in terms of rotor radius $\left(R\right)$ and thickness $\left(t\right)$. Also, the required number of flywheel strings $({N}_{FW})$ for a stable operation is computed from (21).

19 $${E}_{fw}=\frac{1}{2}J{w}_{fw}^{2}$$

20 $${E}_{fw}=\frac{\pi }{4}{\rho }_{r}{R}^{4}{w}_{fw}^{2}t$$

21 $${N}_{FW}=\frac{{E}_{fw}}{{E}_{fwr}}$$

where ${E}_{fw}$ is the required and rated stored kinetic energy in the flywheel $\left(Joule\right)$, ${w}_{fw}$ denotes the rotational angular speed $\left(rad/s\right)$ while $J$ designates the moment of inertia $\left(kg.{m}^{2}\right)$, ${\rho }_{r}$ is the rotor mass density $\left(kg/{m}^{3}\right)$.

### Super capacitors modelling

SCs are characterized by high charging/discharging rates compared to storage batteries. The stored energy in SCs $\left({E}_{SC}\right)$ depends on the capacitance value and applied voltage as revealed in (22). Hereinafter, the power required $\left({P}_{SC}\right)$ of SCs is computed from (23) according to the discharging time $\left({t}_{dis}\right).$ Also, the required number of SCs strings $({N}_{SC})$ for a stable operation is computed from (24).

22 $${E}_{SC}=\frac{1}{2}C{{V}_{SC}}^{2}$$

23 $${P}_{SC}=\frac{{E}_{SC}}{{t}_{dis}}$$

24 $${N}_{SC}=\frac{{P}_{SC}}{{P}_{SCr}}$$

where $C$ is the capacitance value of SC, ${V}_{SC}$ is the applied voltage across the SC terminals, and ${P}_{SCr}$ defines the rated power of the selected SC model.

### Power converter modelling

As it is well known, the generated power from the WT is AC while it is DC from the PV. Moreover, storage batteries are connected through the DC bus while loads may be connected through AC or DC bus. Therefore, bi-directional power converter is used to link between AC and DC buses to execute the rectification or inversion process according to the MG configuration. In this context, the power converter is sized according to (25) knowing the peak load value and converter efficiency.

25 $${P}_{conv}(t)=\frac{{P}_{max}(t)}{{\eta }_{conv}}$$

where ${P}_{conv}(t)$ denotes the required converter power at time t, ${P}_{max}(t)$ signifies the load peak power at time t, and ${\eta }_{conv}$ is the converter efficiency.

## Objective function, associated constraints, and KPIs

HO^@MER^ optimizer [HOMER Pro 3.14.2 https://homerenergy.com/] deploys a modified grid search methodology along with multi-criteria decision analysis to attain the best solution among a set of candidate solutions. It extracts the superior solution with the minimum value of net present cost $(NPC)$or $CoE$, i.e., optimization of configuration and number of renewables/storage units. Independent constraints $\left({N}_{PV}, {N}_{WT}, {N}_{batt}\right)$ in addition to the dependent constraints $\left(EEP\right)$ and capacity shortage factor $({CS}_{f})$ are also fulfilled.

### Objective function

The purpose of the optimization process is to minimize the $CoE$ as explained in (26) by minimizing the $ASC$ which is splitted into three terms as declared in (27).

26 $$OF=Min\left\{CoE\right\}=Min\left\{\frac{ASC}{TASL}\right\}$$

27 $$ASC=ACC+ARC+AOMC-SC$$

where $ACC$ signifies the annual capital cost, $ARC$ denotes the annual replacement cost, $AOMC$ designates the annual operation & maintenance cost, $SC$ is a salvage value, while $TASL$ is the total annual supplied load by the HRMG system.

In this context, $ACC$ is calculated from (28) based on the project initial capital cost $(ICC)$ and capital recovery factor $({CRF}_{(i,ny)})$ which evaluates the money worth as per (29).

28 $$ACC=ICC\times {CRF}_{(i,ny)}$$

29 $${CRF}_{(i,ny)}=\frac{i{\left(1+i\right)}^{ny}}{{\left(1+i\right)}^{ny}-1}$$

30 $$i=\frac{{i}^{i}-f}{1+f}$$

It is worth mentioning that $CRF$ depends on the real interest rate $(i)$ and the project life time in years $(ny)$. The real annual interest rate is calculated form (30) based on the nominal interest rate $({i}^{i})$ and annual inflation rate $(f).$ On the other hand, $ARC$ is calculated from (31) depending on the replacement cost $(RC)$ in addition to the $CRF.$ Moreover, $SC$ is computed from (32) which represents the residual value of the component in the HRMG at the end of project life time.

31 $$ARC={CRF}_{(i,ny)}\times \sum_{nR}\frac{RC}{{\left(1+i\right)}^{tR}}$$

32 $$SC=RC\times \frac{RLT}{CLT}$$

where $tR$ denotes the replacement time in years, $nR$ is a counter for the number of replacements occurred during the project life time, $RLT$ is the component remaining life at the end of the project life span, and $CLT$ is the component life time in years. Since $NPC$ is a cost-effective measure, HRMG configurations may be ranked based on their $NPC$ values as declared in (33). It is calculated from the annual cost saving $ACS$ which is the variance between $ASC$ of the base system and $ASC$ of the proposed HRMG system.

33 $$NPC=\frac{ACS}{{CRF}_{(i,ny)}}$$

Deep look to Eqs. (34), (35), and (36), various forms of cost functions utilized in $ASC$ calculation can be computed based on the set of decision variables. Accordingly, the OF is reformulated comprising the rating of each individual component inside the FCS.

34 $$ICC={ICC}_{WT}\times {P}_{TWT}+{ICC}_{PV}\times {P}_{TPV}+{ICC}_{batt}\times {N}_{batt}+{ICC}_{SC}\times {N}_{SC}+{ICC}_{FW}\times {N}_{FW}+ {ICC}_{conv}\times {P}_{conv}$$

35 $$RC={RC}_{WT}\times {P}_{TWT}+{RC}_{PV}\times {P}_{TPV}+{RC}_{batt}\times {N}_{batt}+{RC}_{SC}\times {N}_{SC}+{RC}_{FW}\times {N}_{FW}+ {RC}_{conv}\times {P}_{conv}$$

36 $$AOMC={AOMC}_{WT}\times {P}_{TWT}+{AOMC}_{batt}\times {N}_{batt}+{AOMC}_{SC}\times {N}_{SC}+{AOMC}_{FW}\times {N}_{FW}$$

It is worth noting that the optimized variables of DGs are the total output power while they are number of strings in the case of ESDs.

### Problem constraints

Set of inequality constraints are fulfilled to attain feasible solutions as indicated in (37)–(42). All optimized decision variables are bounded between lower and upper limits which are deemed as inputs to the optimizer. Moreover, the ESD $SoC$ at any time during charging or discharging processes shall also be between minimum and maximum operating limits to prolong its life time as indicated in (43).

37 $${P}_{TPVmin}\le {P}_{TPV}\le {P}_{TPVmax}$$

38 $${P}_{TWTmin}\le {P}_{TWT}\le {P}_{TWTmax}$$

39 $${N}_{battmin}\le {N}_{batt}\le {N}_{battmax}$$

40 $${N}_{SCmin}\le {N}_{SC}\le {N}_{SCmax}$$

41 $${N}_{FWmin}\le {N}_{FW}\le {N}_{FWmax}$$

42 $${P}_{convmin}\le {P}_{conv}\le {P}_{convmax}$$

43 $${SoC}_{min}\le SoC(t)\le {SoC}_{max}$$

where ${P}_{TPVmin}, {P}_{TPVmax}$, ${P}_{TWTmin}, {P}_{TWTmax}$, ${P}_{convmin},$ and ${P}_{convmax}$ represent the minimum and maximum values of the total output power from PV modules, WT modules, and converter respectively. ${N}_{battmin}, {N}_{battmax}, {N}_{SCmin}, {N}_{SCmax}, {N}_{FWmin},$ and ${N}_{FWmax}$ represent the lower and upper limits of battery strings, SC strings, and flywheel strings, respectively.

### Key performance indicators

Optimal solutions are also evaluated by KPIs which are classified into technical and economical indices that quantify the quality of the solution. Among these technical indicators is the ${UML}_{f}$ which is calculated from (44) that expresses the total amount of demand that are not be supplied during the year. Furthermore, $EEP$ is calculated through (45) which expresses the excess energy that shall be dumped to a thermal load as it cannot be employed to supply the original load or even charge the ESDs.

44 $${UML}_{f}=\frac{{E}_{UML}}{{E}_{demand}} , {E}_{demand}={E}_{load}+{E}_{def}$$

45 $$EEP=\frac{{E}_{surplus}}{{E}_{production}}$$

46 $${CS}_{f}=\frac{{E}_{CS}}{{E}_{demand}}$$

where ${E}_{UML}, {E}_{surplus},$ and ${E}_{production}$ are the total un-met load, excess electric load, and production energy throughout the year, respectively in $kWh/yr$.

Certainly, ${E}_{demand}$ is the total demand power that shall be provided by the HRMG to the load ${(E}_{load})$ and the deferrable energy ${(E}_{def})$. Moreover, the capacity shortage factor $({CS}_{f})$ is determined through (46) from the yearly energy capacity shortage $({E}_{CS})$ between the required and actual operating capacities. It is worth mentioning that there may be excess electricity on a bus and a capacity shortage on another bus if there is an undersized converter at any time slot.

As a measure of RESs effectiveness, the renewable fraction $\left({R}_{f}\right)$ is evaluated through (47) which indicates the energy fraction generated from RESs delivered to the load. In this context, the renewable penetration factor $\left({R}_{pen}\right)$ which is calculated through (48) refers to the ratio between the generated power from RESs $\left({P}_{ren}\right)$ and the load power $\left({P}_{load}\right)$ at each time slot.

47 $${R}_{f}=1-\frac{{E}_{non-ren}+{H}_{non-ren}}{{E}_{load}+{H}_{served}}$$

48 $${R}_{pen}=\frac{{P}_{ren}}{{P}_{load}}$$

where ${E}_{non-ren},$ and ${H}_{non-ren}$ symbolize the non-renewable electrical and thermal production, respectively, while ${H}_{served}$ is the thermal load served by the year.

Among the various economic indicators, present worth $$\left({P}_{w}\right)$$ in ($) is assessed from (49) which aids in estimating the cash flow current value or a future payment. Afterwards, the annual worth $$\left({A}_{w}\right)$$ in $$\left(\$/yr\right)$$ is calculated from (50) which is the product of $${P}_{w}$$ and $${CRF}_{(i,ny)}.$$

49 $${P}_{w}={F}_{w}{\left(\frac{1}{1+i}\right)}^{{N}_{p}}$$

50 $${A}_{w}={P}_{w}\times {CRF}_{(i,ny)}$$

where ${F}_{w}$ is the future worth, and ${N}_{p}$ is the number of periods.

**Figure 7:**
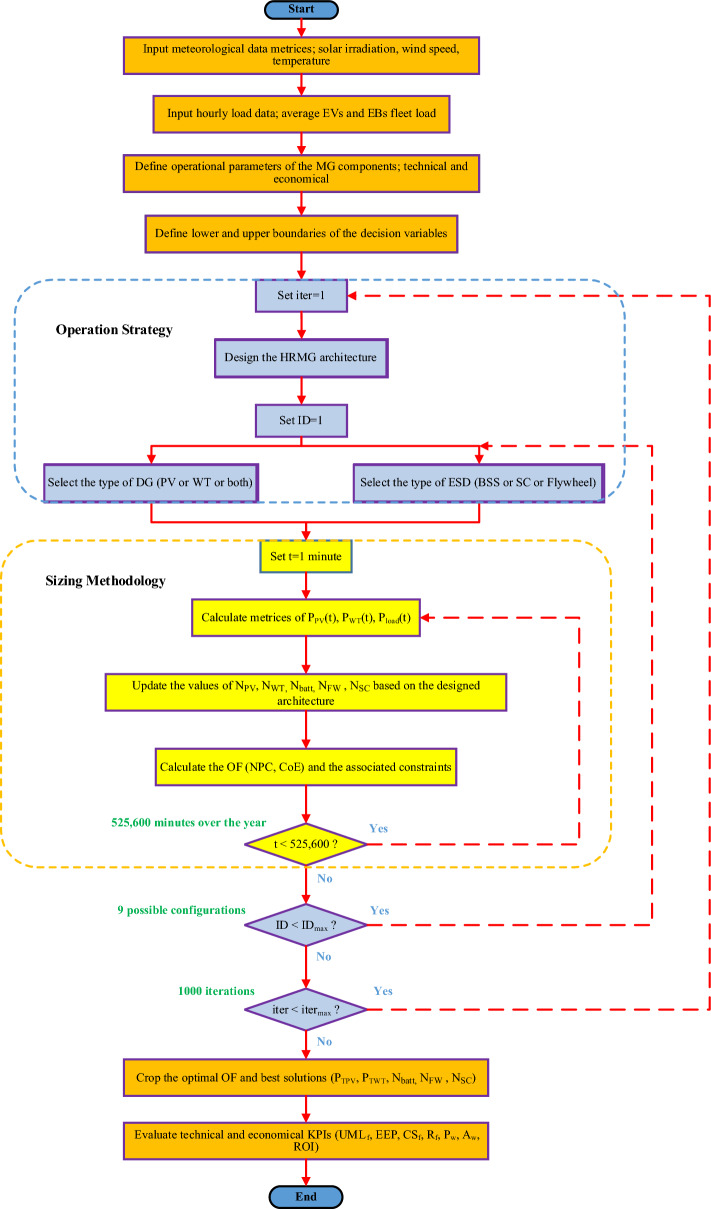
Flowchart of the proposed optimizer.

Another attribute of evaluating the investment’s profitability is the return on investment $\left(RoI\right)$ which gives the ratio between the net income and investment as demonstrated in (51).

51 $$RoI=\frac{\sum_{n}\left({ACF}_{ref}-{ACF}_{cur}\right)}{n\left({ACC}_{cur}-{ACC}_{ref}\right)}$$

where ${ACF}_{ref},$ and ${ACF}_{cur}$ are the annual cash flow of the reference and current system respectively, while ${ACC}_{cur},$ and ${ACC}_{ref}$ are the annual capital cost of the current and reference system respectively. Eventually, the general flow chart of the proposed mathematical model using HO^@MER^ optimizer is demonstrated in Fig. 7. This flowchart illustrates the optimizer’s procedure regarding the operation strategy along with sizing methodology till cropping the final results.

## Simulation results and discussions

### Project portfolio

BRT (Bus Rapid Transit) is a national project organized by the government in Egypt located in the Great Cairo’s Ring Road through 113 km highways. BRT will serve both EBs and EVs fleet across the Ring Road through the expansion from 4 to 7 lanes in each direction^55^. The scope of this research is to design a HRMG comprising PV, WT, BSS, SCs, and flywheel form techno-economic prospective in off-grid configuration. Four dispensers have been dedicated for simultaneous charging of EBs; two of them with rated power of 60 kW and two are 120 kW. Therefore, this research aims at developing a FCS feeding EBs fleet in addition to EVs along the Ring Road to encourage the drivers of private cars to replace their conventional gasoline cars with EVs.

### Meteorological data

**Figure 8:**
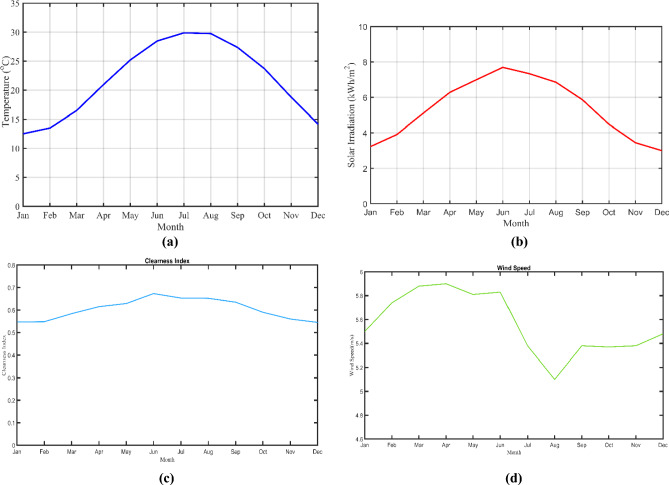
Site monthly meteorological data. (**a**) Temperature, (**b**)  Solar irradiation, (**c**) Clearness index and (**d**)  Wind speed.

The site information (30^0^5.5’N, 31^0^11.8’E) regarding solar irradiation, wind speed, and temperature is obtained from NASA prediction of worldwide energy resources. The average values of solar irradiation, wind speed, and temperature are $5.35\frac{kWh}{{m}^{2}}/day$, $5.56 m/s$, and $21.73^\circ{\rm C}$ respectively in August 2023 based on the selected zone. Moreover, the detailed monthly meteorological data is clarified in Fig. 8.

### EVs and EBs fleet data

**Figure 9:**
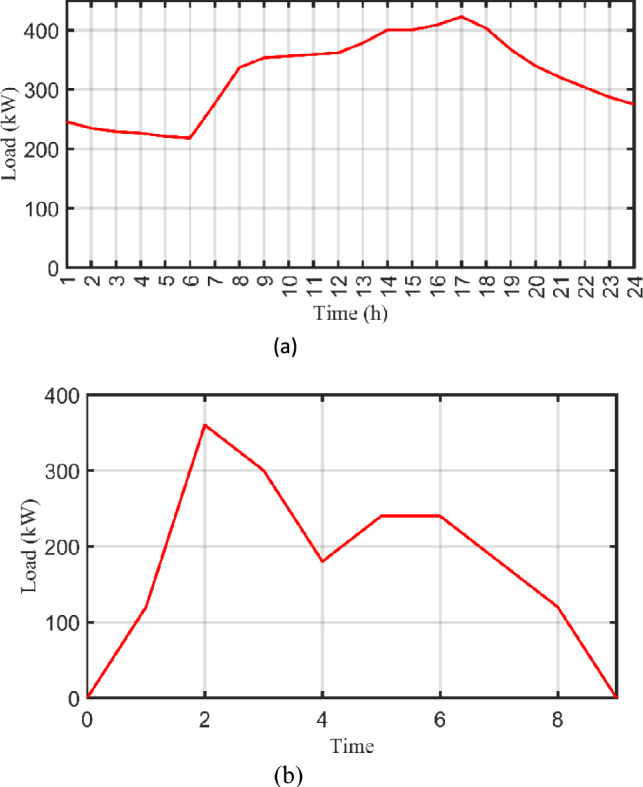
Daily load curve of EVs and EBs fleets across the Ring Road. (**a**) EVs fleet and (**b**) EBs fleet.

Due to the spatial–temporal distribution of EVs, their load data is gathered from a survey of the Cairo’s Ring Road on a typical weekday^56,57^. Cairo’s Ring Road records about 213,000 cars passing through it every day; 80 of them are EVs with various capacities such as 24, 30, and 40 kWh^58^ recorded in 2023. However, EVs fleet data are expected to be doubled in 2040 as reported in^59^ which counts about 500,000 cars with 160 EVs that are included in this research and investigated as the load pattern. Accordingly, the optimized planned model is designed to serve Cairo’s Ring Road during the next 20 years. Moreover, the time congestion effect is considered as shown in Fig. 9a which indicates the peak traffic flow occurs between 2 and 6 pm and other time periods according to the lifestyle in Egypt^60^. Furthermore, the EBs fleet load data is shown in Fig. 9b which demonstrates that the charging process of the EBs fleet occurs between 1 and 8 am^58,59^.

For accurate modelling of fleet load data, random variability factors shall be considered in time step variation and day-to-day variation. ${k}_{tv}$ defines the time step random variability factor, while ${k}_{dv}$ defines the day-to-day random variability factor. By this way, loading profiles of both EVs and EBs will be precisely modelled during the whole year. Based on the nature of the load and studied area, ${k}_{tv}=20 {\%}$, and ${k}_{dv}=20 {\%}$. As it is shown in Fig. 9, the daily peak load of the EVs fleet is about 422.653 kW, average load is about 321.86 kW, and the average energy consumption $({E}_{load})$ per a day is 7724.7 kWh. On the other side, the daily peak load of the EBs fleet is about 360 kW, average load is about 72.5 kW, and the average energy consumption $({E}_{load})$ per a day is 1740 kWh. However, and due to the randomness in time step and daily load variability, the yearly peak load of the EVs fleet is corrected to 792.82 kW while it is about 707.28 kW for the EBs fleet inside the HO^@MER^ optimizer.

### HRMG components specifications

The integration between PV and WT enhances the system performance rather than using only one source in order to cover the shortage in solar irradiation or wind speed. Moreover, and due to the intermittent nature in RESs, ESDs represented in BSSs, flywheels and SCs are investigated. It is worth mentioning that when the generated renewable energy exceeds the load and ESSs are fully charged, the excess energy is used as a dumped load like water heaters. Utilizing fast chargers such as CHAdeMO in addition to flywheels and SCs grant the fast-charging capability to the HRMG due to its deployment in public transportation networks.

**Figure 10:**
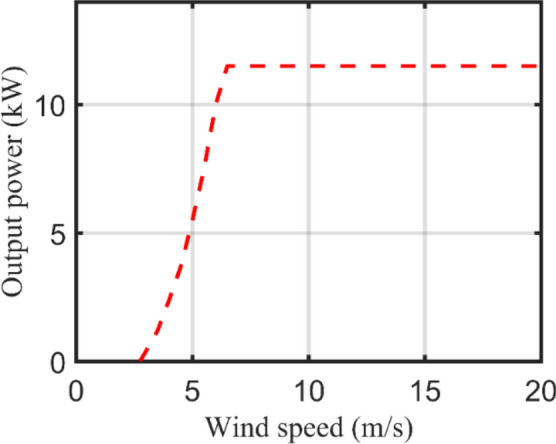
Eocycle 10 kW WT power-speed curve.

**Figure 11:**
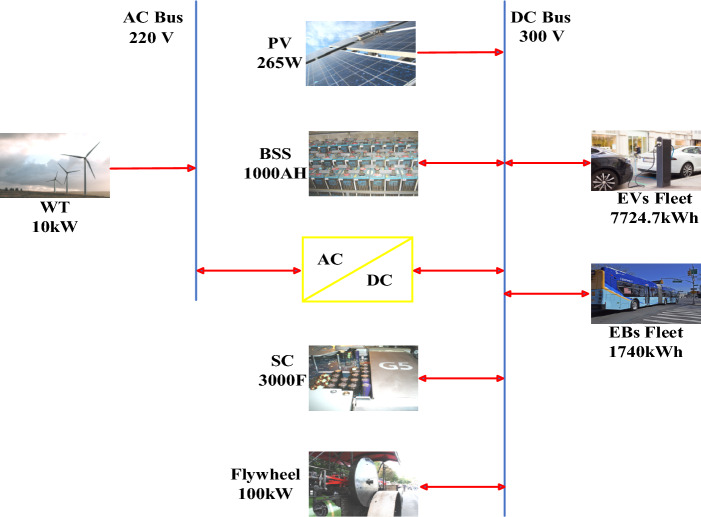
Configuration of the HRMG.

Table 2 lists the technical and economical specifications of PV and WT units as mentioned in^47^ while Fig. 10 displays the actual power-speed curve of the selected WT model. On the other side, Table 3 lists the ESDs specifications including BSSs^47^, flywheels^61^, and SCs^62^. It is worth mentioning that this project has been planned for over 20 years with an annual interest rate of 6% and inflation rate of 2%. Figure 11 demonstrates the HRMG configuration acts as FCS supplying fleets of EVs and EBs with bi-directional power converter connecting AC with DC bus.

**Table 2:** WT and PV specifications.

WT		PV			
Manufacturer	Eocycle	Manufacture	Trina Solar	$${I}_{sc}$$	$$9.1 A$$
$${P}_{r}$$	$$10 kW$$	Type	Polycrystalline	$$ICC$$	$$450 \$/kW$$
$${v}_{r}$$	$$6 m/s$$	$$NOCT$$	$$44^\circ{\rm C}$$	$$RC$$	$$450 \$/kW$$
$${v}_{ci}$$	$$2.75 m/s$$	Efficiency $$@STC$$	$$16.2\%$$	$$AOMC$$	Neglected
$${v}_{co}$$	$$20 m/s$$	$${P}_{mpp}@STC$$	$$265 W$$	Life time	25 years
$${P}_{rmax}$$	$$11.5 kW$$	Temperature coefficient of $${P}_{mpp}$$	$$-0.41 \%/^\circ{\rm C}$$		
$${h}_{h}$$	$$16 m$$	Number of cells	60	**Project Specs**	
$$ICC$$	5,050 $/unit	$${F}_{PV}$$	88%	$$ny$$	20 years
$$RC$$	5,050 $/unit	$${V}_{mpp}$$	$$30.8 V$$	$$i$$	6%
$$AOMC$$	10 $/unit	$${I}_{mpp}$$	$$8.61 A$$	$$f$$	2%
Life time	20 years	$${V}_{oc}$$	$$38.3 V$$		

Significants values are in bold.

**Table 3:** Storage elements and converter specifications.

Batteries		Flywheels		Super Capacitors	
Manufacturer	Hoppecke	Manufacturer	ABB	$$C$$	$$3000 F$$
Type	Lead acid	Charge/discharge capacity	$$100 kW$$	$${V}_{SC}$$	$$3 V$$
$${AHC}_{r}$$	$$1000 AH$$	Energy content	$$25 kWh$$	$${E}_{SC}$$	$$3.75 Wh$$
$${V}_{B}$$	$$2 V$$	$$ICC$$	80,000 $/unit	$$ICC$$	500 $/unit
$$DoD$$	80%	$$RC$$	40,000 $/unit	$$RC$$	500 $/unit
$${E}_{batt,life}$$	$$\text{3,438} kWh$$	$$AOMC$$	1600 $/unit	$$AOMC$$	50 $/unit
$${\eta }_{charge}, {\eta }_{discharge}$$	86%	Life time	20 years	Life time	14 years
$${SoC}_{min}$$	20%			String size	100
$$ICC$$	50 $/unit	**Converter**			
$$RC$$	50 $/unit	$${\eta }_{conv}$$	85%		
$$AOMC$$	5 $/unit	$$ICC$$	$$110 \$/kW$$		
Life time	5 years	$$RC$$	$$110 \$/kW$$		
String size	150	Life time	20 years

### Results of basic model

It is worth mentioning that one minute is considered as the time step in the iteration process that results in total time steps per year of 525,600. Consequently, the problem complexity intensifies, however, it is necessary for the accurate simulation of fast charging/discharging rates of flywheels and SCs. Moreover, the maximum value of ${CS}_{f}(\%)$ during the year is 20% which represents a feasible value in this study to imitate V2G and vehicle to vehicle (V2V) technologies. The optimizer is executed 1000 times with the specified lower and upper boundaries of decision variables as announced in Table 4. The FCS comprises two DGs i.e., PV and WT in addition to three ESDs i.e., BSSs, SCs, and flywheels with the bidirectional power converter. The sizing of various FCS configurations listed in Table 5 results in nine architectures ranked in ascending order regarding the OF and cost values as listed in Table 6. It is worth noting that the optimum values of PV and WT units are reported in kW (total output power) while the optimum values of ESDs are reported in number of units.

**Table 4:** Lower and upper limits of the optimized variables.

PV		WT		Converter		BSS		SC		Flywheel	
${P}_{TPVmin}$	${P}_{TPVmax}$	${P}_{TWTmin}$	${P}_{TWTmax}$	${P}_{convmin}$	${P}_{convmax}$	${N}_{battmin}$	${N}_{battmax}$	${N}_{SCmin}$	${N}_{SCmax}$	${N}_{FWmin}$	${N}_{FWmax}$
0	6000 kW	0	17,000 kW	0	1500 kW	0	25	0	5	0	30

**Table 5:** Various optimized configurations of the HRMG.

Configuration												
ID	Architecture						Sizing					
	PV	WT	BSS	SC	Flywheel	Converter	PV (kW)	WT (kW)	BSS (#)	SC (#)	Flywheel (#)	Converter (kW)
**1**		**✔**	**✔**			**✔**		**870**	**11**			**692**
2	✔		✔				2,031		22			
3	✔	✔	✔			✔	2,005	70	22			469
**4**		**✔**			**✔**	**✔**		**4330**			**14**	**672**
5	✔	✔		✔		✔	3,403	6040		1		937
6	✔	✔				✔	4,037	5950				941
7	✔	✔			✔	✔	2,891	7230			1	786
8		✔		✔		✔		14,330		5		1275
9		✔				✔		17,040				1076

Significant values are in bold.

**Table 6:** $OF$ and cost values for various configurations.

Configuration ID	$ICC(\$)$	$RC(\$)$	$O\&M(\$)$	$SC(\$)$	$NPC(\$)$	$CoE(\$/kWh)$	$AOMC(\$/yr)$
**1**	**597,986.36**	**170,552.03**	**124,809.04**	**0**	**893,347.430**	**0.02243**	**21,582.52**
2	1,079,062.5	341,104.06	225,805.83	84,701.68	1,561,271	0.03911	35,235.74
3	1,103,209.38	341,104.06	226,763.79	83,615.76	1,587.462	0.03969	35,385.09
**4**	**3,380,599.78**	**0**	**365,805.44**	**0**	**3,746,405**	**0.09543**	**26,730.00**
5	4,734,750.38	29,180.34	151,084.63	155,150.98	4,759,864	0.11670	1835.12
6	4,924,876.41	0	81,426.95	168,335.34	4,837,968	0.11850	6350.54
7	5,118,441.67	0	120,840.33	120,537	5,118,745	0.12500	22.16
8	7,626,892.63	145,901.68	538,238.98	66,189.35	8,244,844	0.21030	45,154.71
9	8,723,600.54	0	233,195.84	0	8,956,796	0.22800	17,040.00

Significants values are in bold.

As it is clear that architecture no. 1 is the best candidate architecture which attains $$NPC$$ of 893,347.43 $ and $$CoE$$ of 0.02243 $$\$/kWh$$. This architecture includes WT of 870 kW, converter of 692 kW, and 11 strings of the selected battery model. However, this architecture is accepted only in normal charging mode that lasts for few hours as it doesn’t contain SC or flywheel which simulate the fast-charging process. Therefore, architecture no. 4 is the nominated one in fast charging operation which comprises WT of 4330 kW, converter of 672 kW, and 14 strings of the flywheel selected model (1400 kW). Although there are other fast charging architectures such as no. 5, no. 7, and no. 8, the winner is no. 4 which achieves the lowest cost values i.e., $$NPC$$ of 3,746,405 $ and $$CoE$$ of 0.09543 $$\$/kWh$$. It can be concluded that the fast-charging architecture is about 4 times costly compared to the normal charging architecture which serves about 2,868,735 $$kWh/yr$$.

**Figure 12:**
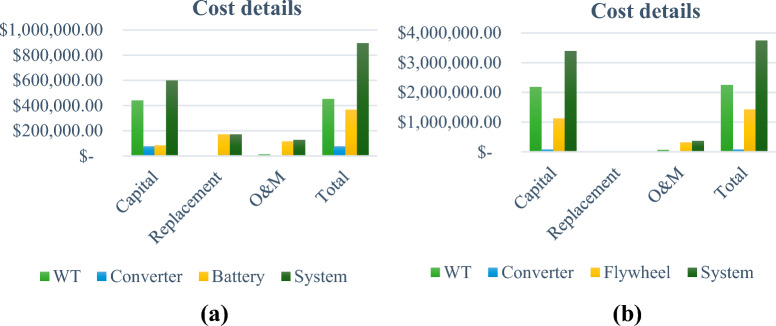
Components cost details for winner architectures. (**a**) Normal charging and (**b**) Fast charging.

Afterwards, technical and economical KPIs are evaluated for each scenario and tabulated in Table 7 and Table 8 respectively. As it is observed, architecture no. 1 accomplishes $${UML}_{f}$$ of 15.8% and $$EEP$$ of 30.8% while the elected fast charging architecture accomplishes $${UML}_{f}$$ of 17% and $$EEP$$ of 86.6%. on the other side, it attains a $${P}_{w}$$ of 2,853,058 $, $${A}_{w}$$ of 208,478 $/yr, and $$RoI$$ of − 5.2%. Furthermore, technical and economical KPIs are estimated also for the other architectures, however, the selected architectures are the optimal from economic perspective. In this context, capital, replacement, operation & maintenance (O&M), and total costs of the system components in addition to the whole architecture are depicted in Fig. 12 for normal and fast charging techniques.

**Table 7:** Technical KPIs for various configurations.

Configuration ID	${UML}_{f}(\%)$	$EEP(\%)$	${CS}_{f}(\%)$	${R}_{f}(\%)$	${R}_{pen}(\%)$
**1**	**15.8**	**30.8**	**19.8**	**100**	**175**
2	15.6	11	20.1	100	121
3	15.4	19.1	19.9	100	134
**4**	**17**	**86.6**	**20.1**	**100**	**866**
5	13.7	92.2	20.1	100	1388
6	13.6	92.3	20.1	100	1406
7	13.4	93.1	18.5	100	1586
8	17.1	96	20.1	100	2936
9	16.9	96.6	20.1	100	3483

Significants values are in bold.

**Table 8:** Economical KPIs for various configurations.

Configuration ID	${P}_{w}(\$)$	${A}_{w}(\$/yr)$	$RoI(\%)$
**1**	**0**	**0**	**0**
2	667,923	48,806	− 7.2
3	694,114	50,720	− 7.1
**4**	**2,853,058**	**208,478**	**− 5.2**
5	3,866,517	282,533	− 4.4
6	3,944,621	288,240	− 4.2
7	4,225,398	308,757	− 4.4
8	7,351,497	537,186	− 5.3
9	8,063,449	589,209	− 4.9

Significants values are in bold.

### Results with resources uncertainty

Hereinafter, the optimization problem gets more sophisticated by introducing uncertainties in renewable resources such as solar irradiation, wind speed, and ambient temperature. The uncertainty range is bounded between − 10% and + 10% of the measured resources as indicated Fig. 8 which results in 27 probable study cases. HO^@MER^ optimizer follows the spider graph approach for modelling the uncertainties in performing the sensitivity analysis. In this context, only the winning configurations are mentioned in Table 9 either in normal or fast charging operation to avoid the lengthening of the paper. Accordingly, and irrespective of the uncertainty values, the winner configuration is the normal charging is WT/BSS/converter while the winner one in fast charging mode is PV/WT/flywheel/converter with some cases in which the PV is not included in the solution. As it is observed in the results, some uncertainty conditions have negligible effect on the FCS architecture such as study case no. 2, 5, 8, 11, and more.

**Table 9:** Winner configurations considering resources uncertainty.

Study case	Resources uncertainty			Architecture						
				Normal			Fast charging			
	${G}_{av}$	${T}_{amb}$	${V}_{an}$	WT (kW)	BSS (#)	Converter (kW)	PV (kW)	WT (kW)	Flywheel (#)	Converter (kW)
1	− 10%	− 10%	− 10%	1020	15	722	-	5220	27	849
2	− 10%	− 10%	0	870	11	692	-	4330	14	672
3	− 10%	− 10%	+ 10%	750	8	768	2321	2260	2	985
4	− 10%	0	− 10%	1020	15	722	-	5220	27	849
5	− 10%	0	0	870	11	692	-	4330	14	672
6	− 10%	0	+ 10%	750	8	768	2317	2270	2	979
7	− 10%	+ 10%	− 10%	1020	15	722	-	5220	27	849
8	− 10%	+ 10%	0	870	11	692	-	4330	14	672
9	− 10%	+ 10%	+ 10%	750	8	768	2412	2260	2	990
10	0	− 10%	− 10%	1020	15	722	-	5220	27	849
11	0	− 10%	0	870	11	692	-	4330	14	672
12	0	− 10%	+ 10%	750	8	768	1116	2300	3	986
13	0	0	− 10%	1020	15	722	-	5220	27	849
14	0	0	0	870	11	692	-	4330	14	672
15	0	0	+ 10%	750	8	768	816	2250	4	1096
16	0	+ 10%	− 10%	1020	15	722	5650	3030	6	787
17	0	+ 10%	0	870	11	692	-	4330	14	672
18	0	+ 10%	+ 10%	750	8	768	807	2260	4	1095
19	+ 10%	− 10%	− 10%	1020	15	722	-	5220	27	849
20	+ 10%	− 10%	0	870	11	692	-	4330	14	672
21	+ 10%	− 10%	+ 10%	750	8	768	1577	1700	4	930
22	+ 10%	0	− 10%	1020	15	722	-	5220	27	849
23	+ 10%	0	0	870	11	692	1951	2380	5	757
24	+ 10%	0	+ 10%	750	8	768	1339	1790	4	1058
25	+ 10%	+ 10%	− 10%	1020	15	722	-	5220	27	849
26	+ 10%	+ 10%	0	870	11	692	1979	2340	5	818
27	+ 10%	+ 10%	+ 10%	750	8	768	1343	1790	4	1055

**Figure 13:**
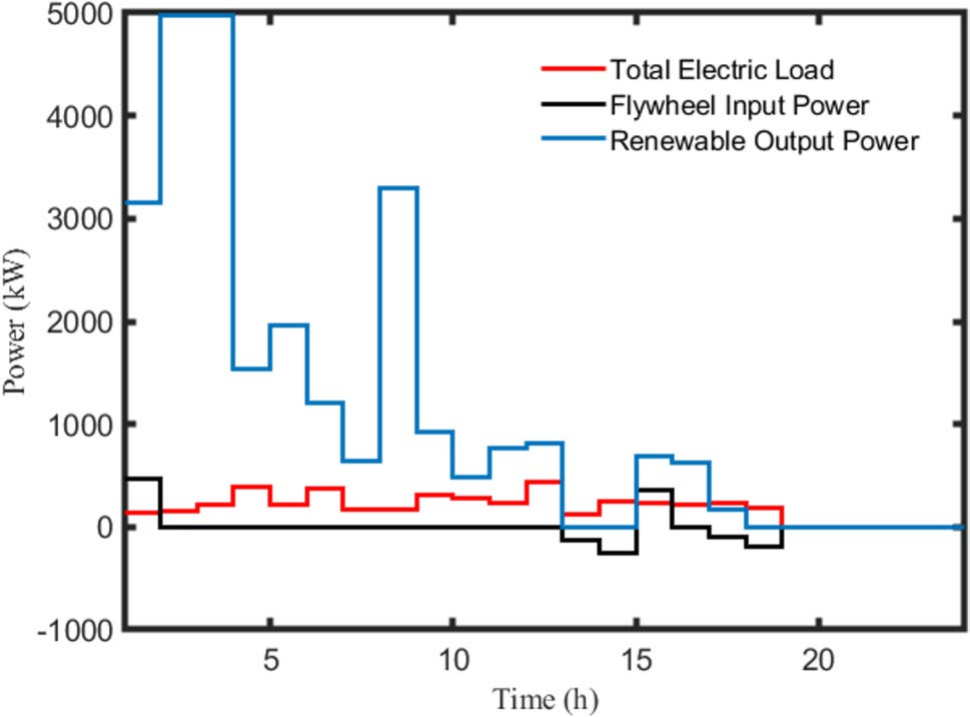
Hourly power analysis of a random day for fast charging mode.

Figure 13 depicts the hourly power analysis of a random day for fast charging operation. Obviously, the total electrical demand is always met by the WT power or the storage power inside the flywheel. This curve demonstrates the feasibility of the nominated configuration during fast charging due to the low energy density of the flywheel. It can be noticed that when the renewable output power is zero at 13:00 and 14:00, the flywheel can be utilized to charge the EVs loads in quick mode before its energy is fully dissipated. Consequently, the high-power density of the flywheel is exploited in fast charging operation while there is no obstacle regarding the low energy density in continuing the charging operation.

**Figure 14:**
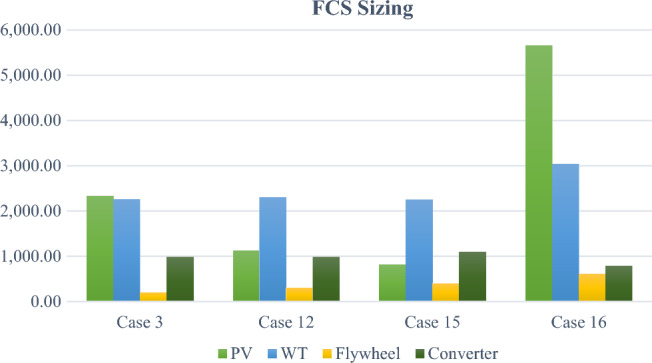
Components sizing for fast charging mode considering uncertainty.

However, some study cases involve huge variations to the original FCS configuration like case no.3 which comprises PV of 2321 kW, WT of 2260 kW, 2 strings of flywheels, and 985 kW converter. Nevertheless, this architecture requires $$NPC$$ of 2,432,078 $ and $$CoE$$ of 0.06003 $$\$/kWh$$ as indicated in Table 10 which achieves a notable reduction compared to the original configuration of 35% and 37% in $$NPC$$ and $$CoE$$ respectively. It is worth mentioning that this huge divergence results from eminent decrement in solar irradiance and temperature by 10% and increment in wind speed by 10% also. When solar irradiation and wind speed increase by 10%, the outcome solution engenders the most economic configuration fulfilling $$NPC$$ of 1,999,018 $ as demonstrated in case no. 24. This is an anticipated conclusion as by increasing solar and wind resources, the required installed components will be minified as well. Last but not least, Fig. 14 shows the sizing of the FCS installed capacity of arbitrary selected architectures. Outspokenly, this optimization process requires about 25 h of PC operation which entices the attraction for implementing the methodology discussed in the next section.

**Table 10:** Performance assessments of fast charging mode considering resources uncertainty.

Case	$NPC (\$)$	$CoE (\$/kWh)$	$AOMC (\$/yr)$	${UML}_{f} (\%)$	$EEP (\%)$	${CS}_{f} (\%)$	${P}_{w} (\$)$	${A}_{w} (\$/yr)$	$RoI (\%)$
1	5,552,096	0.142	48,420	17.3	87.5	20.1	4,444,562	324,771	− 5.5
2	3,746,405	0.09543	26,730	17.0	86.6	20.1	2,853,058	208,478	− 5.2
3	2,432,078	0.06003	1612	14.3	82.2	20.1	1,692,385	123,665	− 3.9
4	5,552,096	0.14200	48,420	17.3	87.5	20.1	4,444,562	324,771	− 5.5
5	3,746,405	0.09543	26,730	17.0	86.6	20.1	2,853,058	208,478	− 5.2
6	2,434,917	0.06010	1589	14.3	82.2	20.1	1,695,224	123,873	− 3.9
7	5,552,096	0.14200	48,420	17.3	87.5	20.1	4,444,562	324,771	− 5.5
8	3,746,405	0.09543	26,730	17.0	86.6	20.1	2,853,058	208,478	− 5.2
9	2,469,825	0.06095	1890	14.3	82.3	20.1	1,730,133	126,424	− 3.9
10	5,552,096	0.14200	48,420	17.3	87.5	20.1	4,444,562	324,771	− 5.5
11	3,746,405	0.09543	26,730	17.0	86.6	20.1	2,853,058	208,478	− 5.2
12	2,062,804	0.05122	3699	14.8	80.6	20.1	1,323,111	96,682	− 4.1
13	5,552,096	0.14200	48,420	17.3	87.5	20.1	4,444,562	324,771	− 5.5
14	3,746,405	0.09543	26,730	17.0	86.6	20.1	2,853,058	208,478	− 5.2
15	2,028,338	0.05059	6164	15.2	79.5	20.1	1,288,646	94,163	− 4.2
16	4,576,540	0.11580	4586	16.4	87.8	20.1	3,469,005	253,486	− 3.9
17	3,746,405	0.09543	26,730	17.0	86.6	20.1	2,853,058	208,478	− 5.2
18	2,029,598	0.05064	6202	15.2	79.5	20.1	1,289,905	94.255	− 4.2
19	5,552,096	0.14200	48,420	17.3	87.5	20.1	4,444,562	324,771	− 5.5
20	3,746,405	0.09543	26,730	17.0	86.6	20.1	2,853,058	208,478	− 5.2
21	2,035,415	0.05081	3295	15.3	77.0	20.1	1,295,723	94,681	− 4
22	5,552,096	0.14200	48,420	17.3	87.5	20.1	4,444,562	324,771	− 5.5
23	2,623,701	0.06591	4436	15.8	82.1	20.1	1,730,353	126,440	− 4
24	1,999,018	0.04989	4111	15.3	77.1	20.1	1,259,326	92,021	− 4
25	5,552,096	0.14200	48,420	17.3	87.5	20.1	4,444,562	324,771	− 5.5
26	2,621,304	0.06588	4309	15.8	81.8	20.1	1,727,957	126,265	− 4
27	2,000,455	0.04994	4097	15.3	77.1	20.1	1,260,762	92,126	− 4

### Deep learning radial basis network

#### Initiation

The computational time of HO^@MER^ optimizer is about 50 min for each individual run which is logic due to the high complexity in the optimization process. This is due to the fact of utilizing 525,600-time steps per year to emulate the ultra-discharging performance of flywheels and SCs. However, outcome results from the optimization model are crucial for the training purposes of the upcoming methodology.

In this subsection, one of deep learning toolboxes in MATLAB environment called radial basis network (RBN) is implemented. Deep learning RBN differs from the traditional feed forward neural network in that it requires more neurons and can be designed and trained in a fraction of time. In this paper, RBN is exploited for predicting the optimal sizing of FCS components with variations in resources availability, fleet loading, in addition to technical and economical KPIs. Moreover, it can be used as online energy management strategy inside the FCS as it takes only few seconds compared to the HO^@MER^ optimizer. The RBN passes through 4 stages as follows:

#### Step 1: RBN design

The RBN can be designed as indicated in (52) using the $newrb$ command by defining the input vector $P$ and output vector $T.$ Furthermore, the targeted mean square error (MSE) is also defined in the parameter $goal$, while $spread$ designates for the spread in radial basis function, $MN$ denotes the maximum number of neurons, and $DF$ denotes the number of neurons to be added between displays. It is worth mentioning that the larger the $spread$ is, the smoother the function approximation. However, too many neurons are required for this purpose for fast charging function to create a generalized RBN.

52 $$net=newrb\left(P,T,goal,spread,MN,DF\right)$$

**Figure 15:**
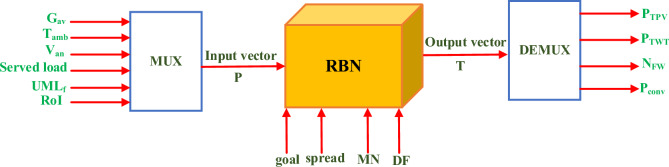
RBN architecture with adjusting parameters.

Figure 15 demonstrates the architecture of RBN with the corresponding adjusting parameters. It can be observed that the multiple inputs pass through MUX to unify them to a single input matrix to the RBN. Moreover, the output vector is split into the targeted output values through the DEMUX.

In this context, the specified values of the design parameters for an accurate design are: $goal=0$, $spread=\text{30,000}$, $MN=\text{1,000},$ and $DF=\text{1,000}.$ It is worth mentioning that the selection of these values are determined after diverse trials till the least error is attained. However, these values may be changed in different problems.

#### Step 2: RBN training

The RBN is trained using the obtained optimized solutions considering resources uncertainty form the previous section. In this regard, 25 set of data comprising 6 input vectors as follows:

${G}_{av}$, ${T}_{amb}$, ${V}_{an}$, served load, ${UML}_{f},$ and $RoI$, and 4 output vectors as follows: PV, WT, flywheel, and converter sizing are considered. Thus, the dimension of $P$ and $T$ matrices are 6 × 25 and 4 × 25 respectively.

#### Step 3: RBN validation

**Figure 16:**
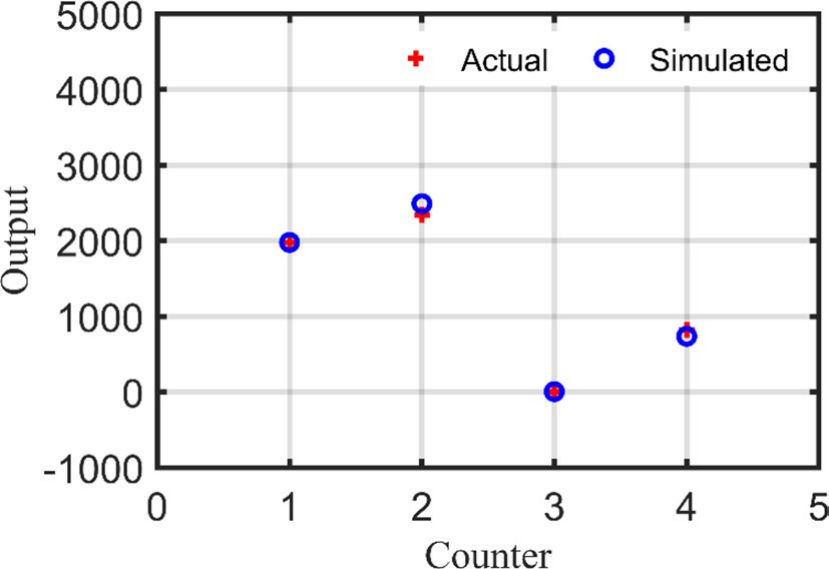
RBN validation using study case no. 26.

**Figure 17:**
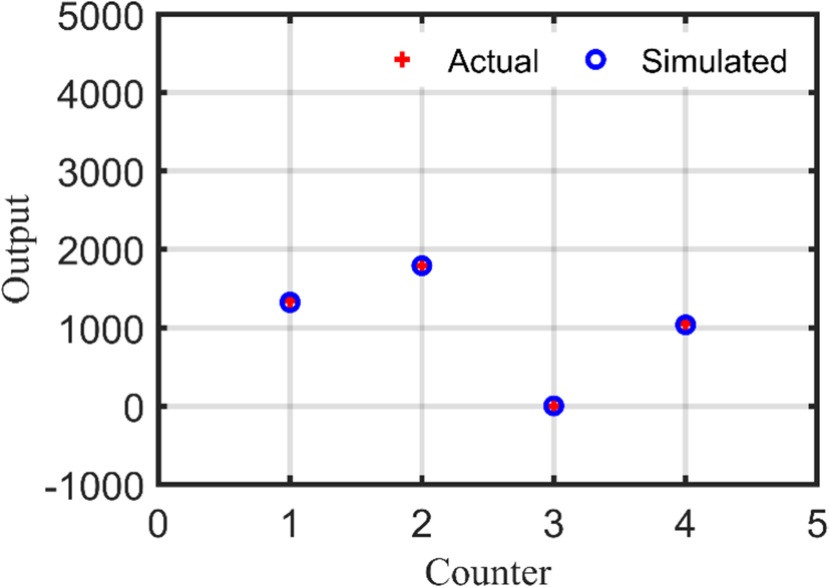
RBN validation using study case no. 27.

In this stage, RBN performance is validated using two study cases from Table 9 in which the simulated output is compared to the actual output for each study case. In addition, errors in per unit (PU) are calculated for each output as demonstrated in Table 11 and Table 12 for study case no. 26 and no. 27 respectively. Furthermore, Fig. 16 depicts the deviations between actual and simulated output using RBN for case no. 26 while Fig. 17 shows the deviations for case no. 27. Consequently, mean absolute error (MAE) and MSE are computed for each study case as follows:

- Study case no. 26: MAE = 0.0441, MSE = 0.003600.
- Study case no. 27: MAE = 0.0077, MSE = 0.000106.

**Table 11:** Validation assessment of the RBN for study case no. 26.

Unit	Actual output	Simulated output	Error (PU)
PV (kW)	1979	1977	0.001
WT (kW)	2340	2489	0.064
Flywheel (#)	5	5	0.011
Converter (kW)	818	736	0.101

**Table 12:** Validation assessment of the RBN for study case no. 27.

Unit	Actual output	Simulated output	Error (PU)
PV (kW)	1343	1324	0.014
WT (kW)	1790	1788	0.0009
Flywheel (#)	4	4	0
Converter (kW)	1055	1039	0.015

#### Step 4: RBN operation

Eventually, the RBN is used for an online energy dispatch strategy to find the optimal output power from each component inside the FCS as explained in Table 13. Various operational scenarios along with altering in geographical conditions are established to determine the optimal solution. It can be noted that when + 5% increase in solar irradiance, temperature, and wind speed, while the served load increased by 2%, the FCS comprises 1829 kW PV, 1813 kW WT, and 4 flywheels. Furthermore, when the temperature decreases by 5% and wind speed increases by 5%, the online dispatch controller manages the charging power between PV and WT at 1679 kW and 5587 kW respectively with 14 strings of flywheels.

**Table 13:** Energy management of FCS components using RBN.

${G}_{av}$	${T}_{amb}$	${V}_{an}$	Served load	${UML}_{f}(\text{\%})$	$RoI(\text{\%})$	PV (kW)	WT (kW)	Flywheel (#)	Converter (kW)
+ 5%	+ 5%	+ 5%	+ 2%	15.3	− 4	1829	1813	4	1294
+ 6%	+ 6%	+ 4%	0	10	− 4	1033	4782	14	1421
+ 2%	+ 3%	+ 3%	+ 1%	3	− 3	1244	6165	6	1437
+ 7%	+ 6%	− 2%	+ 1%	15.3	− 4	3067	3318	6	489
− 2%	0	0	+ 1%	5	− 3	2304	5426	6	1465
0	− 5%	+ 5%	0	3	− 3	1679	5587	14	2216
0	+ 4%	− 4%	+ 1%	15.3	− 4	3265	3348	6	613
− 3%	− 4%	0	0	15.3	− 4	3434	2835	13	1297
+ 2%	− 8%	− 5%	+ 2%	0	0	10,068	418	3	4123
− 2%	0	+ 2%	+ 2%	0	0	9398	512	3	3922

KPIs effect is tackled through enforcing ${UML}_{f}$ and $RoI$ to be 0% which in turns grants the dominance to the PV units with rated power of 10,068 kW or 9398 kW based on the operation scenario mentioned in Table 13. Moreover, 3 strings of flywheels are required to achieve this condition.

In fact, RBN harvests the optimal result in about 3 s which is very lower than the computational time of the optimizer. Despite the technical benefits of the proposed methodology, there are some issues and limitations that have to be mentioned. First, LPSP index is not included into the optimizer’s mechanism, however, it can be compensated by other factors such as ${UML}_{f}$ and ${CS}_{f}$. Additionally, the RBN parameters shall be well-tuned to guarantee the result’s accuracy.

## Conclusions

With the help of deep learning RBN, this study is a fresh attempt at an online energy management dispatch approach for FCS. Along Cairo’s Ring Road, initial loads of both EV and EB fleets are defined, along with an evaluation of renewable resources. Then, with relation to the NPC and CoE, all feasible FCS configurations are rated in ascending order. It has been determined that the charging station’s ideal architecture depends on whether it will function in standard or rapid charging mode. Therefore, it has been established that choosing the PV/WT/flywheel/converter design is the ideal setup for quick charging operation. The winning charging architecture costs nearly four times as much as the standard charging architecture and consists of a WT of 4330 kW, a converter of 672 kW, and 14 strings of the flywheel. As a result, there are several variables related to renewable resources, such as sun irradiance, temperature, and wind speed, that can affect how well this ideal design performs. Finally, the RBN is put into use for the online energy management strategy, validated with the optimal outcomes attained, and executed using different operating situations. This research area is still being looked into, though, because the FCS operation in on-grid mode necessitates greater attention from a techno-economic standpoint.

## Data Availability

The datasets generated and/or analyzed during the current study are not publicly available due to intellectual property rights but are available from the corresponding author on reasonable request.
